# Broadband Visible Nonlinear Absorption and Ultrafast Dynamics of the Ti_3_C_2_ Nanosheet

**DOI:** 10.3390/nano10122544

**Published:** 2020-12-17

**Authors:** Yabin Shao, Chen Chen, Qing He, Wenzhi Wu, Chensha Li, Yachen Gao

**Affiliations:** 1 Electronic Engineering College, Heilongjiang University, Harbin 150080, China; shao_yabin@163.com (Y.S.); wuwenzhi@hlju.edu.cn (W.W.); 2 Department of Computer & Electrical Engineering, East University of Heilongjiang, Harbin 150086, China; rukawa1600@163.com; 3 Collaborative Innovation Center of Steel Technology, University of Science and Technology Beijing, Beijing 100083, China; 123simon@163.com; 4 School of Chemistry and Material Sciences, Heilongjiang University, Harbin 150080, China; 2015068@hlju.edu.cn

**Keywords:** nonlinear absorption, transient absorption, 2D material, MXene

## Abstract

The Ti_3_C_2_ nanosheet, as a new two-dimensional (2D) group, has been found to have attractive characteristics as material for electromagnetic shielding and energy storage. In this study, the nonlinear broadband absorption and ultrafast dynamics of the Ti_3_C_2_ nanosheet were investigated using nanosecond open-aperture Z-scan and transient absorption techniques. The mechanism of two-photon absorption (TPA) was revealed in the visible region (475–700 nm). At lower incident energies, nonlinear absorption could not happen. When the laser energy increased to 0.64 GW/cm^2^, electrons in the valence band could absorb two photons and jump to the conduction band, with TPA occurring, which meant that the sample exhibited reverse saturable absorption (RSA). In addition, when transient absorption was used to investigate the ultrafast carrier dynamics of the sample, it demonstrated that the relaxation contains a fast decay component and a slow one, which are obtained from electron–phonon and phonon–phonon interactions, respectively. Moreover, with the increasing pump fluence, the fast decay lifetime τ_1_ increased from 3.9 to 4.5 ps, and the slow one τ_2_ increased from 11.1 to 13.2 ps. These results show that the Ti_3_C_2_ nanosheet has potential applications in broadband optical limiters.

## 1. Introduction

Ever since the discovery of graphene [1], two-dimensional (2D) materials have attracted much interest due to their novel electronic, optical, and mechanical properties which are different from those of their bulk forms. As the first 2D material identified, graphene has been intensively studied for various applications, such as optical modulators [2], ultrafast laser generation [3], and surface plasmonic [4], whereas the gapless Dirac-cone band structure limits its possibilities for applications [5]. Recently, a group of new 2D materials, which show the semiconductor and metallic phase, have provided evidence of attractive nonlinear optical (NLO) properties different from those of organic materials [6,7,8]. For example, topological insulators (TIs), transition metal dichalcogenides (TMDCs), and black phosphorus (BP), are the best materials in ultrafast fiber lasers mode lock [9]. However, the inherent lack of fine-controlled material and a manufacturing process still remains a challenge. The development of novel promising 2D phase NLO materials is still a long-term objective. Recently, MXene [5], as an emerging branch of the 2D material race, has been shown to exhibit tempting merits, such as a good conductivity, high elastic moduli, a high electric capacity, a tunable bandgap, and a high optical transparency [10,11,12,13,14]. By taking advantage of the linear optical properties, experimental and theoretical research on Ti_3_C_2_ nanomaterials is developing rapidly [15,16,17,18,19,20]. Due to its attractive features, MXene has become a new hotspot for research. The NLO properties of Ti_3_C_2_ continue to attract attention [21].

In the last three years, many scholars have made great breakthroughs in the NLO properties of Ti_3_C_2_. For example, in 2018, Jiang et al. reported the NLO properties of Ti_3_C_2_ measured by the Z-scan technique excited by a 100 fs pulse at the wavelengths of 800, 1064, 1550, and 1800 nm in open aperture (OA) and closed aperture (CA). The nonlinear refractive index and the real part of the third-order nonlinear optical susceptibility were revealed [22], demonstrating the value of NLO application to infrared bands. Results obtained through 7 ns laser pulses at 800, 1064 [23], and 1560 nm [24] found that the plasmon-induced saturable absorption (SA) behavior increased in the ground state absorption at phonon energies above the threshold for free carrier oscillations. Additionally, MXene, as a saturable absorber, was successfully used for the first time [25]. In the successive year of 2019, the SA properties of Ti_3_C_2_ were measured by an OA Z-scan excited by 640, 800, 1064, 1550, 1560 [26,27,28], and 2800 nm [29], representing a huge expansion for ultrafast photonics applications. In 2020, Li et al. identified the large third-order nonlinear susceptibility through spatial self-phase modulation [30], and an all-optical modulator was fabricated based on the spatial cross-phase modulation effect.

Nevertheless, all of the previous studies were conducted using a single wavelength laser in the infrared and near-infrared spectrum. In fact, the wavelength range available for measurement significantly influences the measurement results. The NLO properties and ultrafast dynamics process are also wavelength-dependent and energy-dependent, but their NLO performance and carrier dynamics are still unknown. To the best of our knowledge, this is the first work to reveal the nonlinear absorption property in the whole visible region. Here, the NLO properties of the Ti_3_C_2_ nanosheet are investigated at a broadband wavelength in the visible spectrum ranging from 475 to 700 nm. Furthermore, the pump fluence-dependent and wavelength-dependent ultrafast dynamics of Ti_3_C_2_ were also studied with femtosecond transient absorption measurements.

## 2. Materials and Methods

### 2.1. Preparation of the Ti_3_C_2_ Nanosheet

The MXene used in this work is Ti_3_C_2_, which was produced by the selective etching of Al from the MAX precursors. The MXene powder was produced by etching with a fluoride salt, which was a mixture solution of hydrochloric acid (HCl) and lithium fluoride (LiF), at 35 °C for 24 h from Ti_3_AlC_2_ (MAX phase) [5]. After etching, the obtained Ti_3_C_2_ flakes were produced by 1 h sonication, followed by 1 h centrifugation at 3500 rpm. Furthermore, methods of manufacturing two-dimensional materials mostly require exfoliation from bulk-layered materials because of their weaker interlayer Van der Waals values [31]. Herein, chemical stripping was performed to obtain multilayered Ti_3_C_2_ nanosheets.

### 2.2. Optical Experimental Setup

#### 2.2.1. Open-Aperture Z-Scan

For the purpose of revealing the NLO absorption properties of Ti_3_C_2_ nanosheet dispersion, open-aperture Z-scan measurements were applied. A 6 ns Q-switched Nd:YAG nanosecond pulse laser (Surelite II, Continuum, San Jose, CA, USA) was used. In order to avoid thermally-induced nonlinearity scattering, the repetition rate of the laser was chosen to be 5 Hz. An optical parametric oscillator (OPO) was used to obtain the lasers with different wavelengths ranging from 475 to 700 nm [32]. The laser beam was focused through a lens (f = 20 cm) on a quartz cuvette (2 mm) filled with Ti_3_C_2_ nanosheet aqueous solution, and the laser beam waist diameter was about ω_0_ = 200 μm. The transmittance signals were precisely detected as the sample moved step by step along the translation stag (TSA200, Zolix, Beijing, China). The Z-scan signals, which are the transmitted pulses of the sample for every z point, were collected by an energy detector (J-10MB-LE, Coherent, Santa Clara, CA, USA) and registered through an energy meter (EPM2000, Coherent, Santa Clara, CA, USA).

#### 2.2.2. Transient Absorption (TA) Spectroscopy

The ultrafast carrier dynamics were revealed by femtosecond transient absorption (TA) spectroscopy measurements at room temperature. The laser pulse (~35 fs, 800 nm, and 1 kHz) was originally generated through a Ti:sapphire regenerative amplifier (Astrella, Coherent, Santa Clara, CA, USA). Furthermore, in order to double the photon energy to 400 nm as the pump beam, a BBO crystal was used. For the purpose of producing a white-light continuum (420 to 750 nm) as a probe beam, a sapphire plate was used. The output probe beam laser was focused on a quartz cuvette (2 mm) filled with Ti_3_C_2_ nanosheets aqueous solution. The transmitted probe and reference beam intensity were detected by two highly sensitive spectrometers (Avantes-950F, Avantes, Apeldoorn, the Netherlands) after the sample relative delay by a stepper motor-driven optical delay line (TSA-200, Zolix, Beijing, China). All of the signals detected by the spectrometers and the trigger of the 500 Hz mechanical chopper were modulated in the lock-in amplifier, and then immediately recorded by the computer. By calibrating the measurement of a standard sample, pump and probe beams overlapped spatially. The group-velocity dispersion of the white-light continuum was verified by the optical Kerr signal from the SiO_2_ substrate [33].

## 3. Results and Discussions

### 3.1. Characterization of the Ti_3_C_2_ Nanosheet

**Figure 1:**
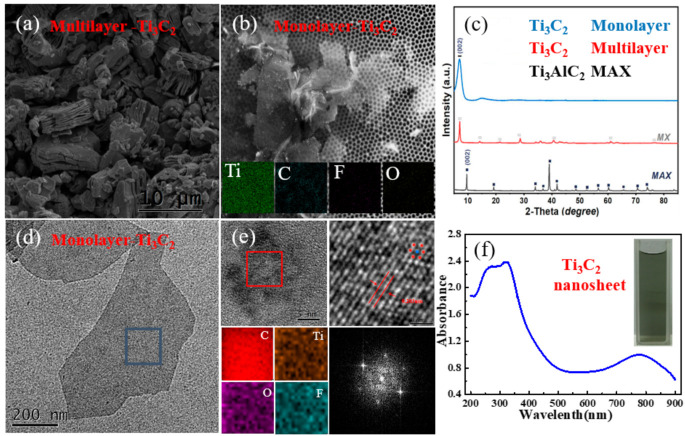
(**a**,**b**) Scanning electron microscopy (SEM) images of multilayer and monolayer Ti_3_C_2_ nanosheets; (**c**) X-ray diffraction patterns of MAX phase Ti_3_AlC_2_ (black), monolayer Ti_3_C_2_ (red), and multilayer Ti_3_C_2_ nanosheets (blue); and (**d**) transmission electron microscopy (TEM) image of a monolayer Ti_3_C_2_ nanosheet. (**e**) The top panels show a high-resolution TEM image and schematic illustration of the atomically resolved image with an overlaid schematic atomic structure, and the bottom panels present STEM-energy-dispersive X-ray (EDX) mapping and Fast Fourier Transform (FFT) of the Ti_3_C_2_ original image. (**f**) Linear absorption spectra of the Ti_3_C_2_ nanosheet, where the inset is a picture of the aqueous solution of the Ti_3_C_2_ nanosheet.

A summary of the morphological multilayered and monolayered features is provided in Figure 1a,b. Microstructures of multilayered and monolayered features were observed by scanning electron microscopy (SEM, ZEISS ULTRA 55, Oberkochen, Germany). In Figure 1a, the scanning electron microscopy (SEM) picture of multilayered flakes with a lateral size of ~0.4 μm is presented. In Figure 1b, a monolayered Ti_3_C_2_ nanosheet with a size of ~1 μm can be observed. In this work, plenty of sheets had a multilayer structure and dispersed uniformly. An organ-like structural surface manifested the successful fabrication of multilayer Ti_3_C_2_ nanosheets. The phase transitions before and after etching were investigated by patterns of X-ray diffraction (XRD, Seifert-FPM, Freiberg, Germany). The observed structure is in harmony with the shift of X-ray diffraction (XRD) results in Figure 1c. XRD measurements of MAX phase Ti_3_AlC_2_ (black), monolayer Ti_3_C_2_ (blue), and multilayer Ti_3_C_2_ nanosheets (red) are shown in Figure 1c. All of the pattern peaks related to Ti_3_AlC_2_ MAX precursors disappeared after the HF etching treatment. Furthermore, the XRD peaks agree with those recorded in the literature. For comparison, low-intensity peaks can be seen in the XRD diffraction patterns of the precursors (black). The post-etching weak peaks (blue and red) can be observed in the XRD image of the Ti and C present in the starting material. In addition, Figure 1c shows a peak shift of the (002) diffraction in monolayer and multilayer Ti_3_C_2_ to a much lower angle relative to that of Ti_3_AlC_2_, reflecting expansion of the interlayer distance. This behavior is attributed to removing the Al layer from the precursor Ti_3_AlC_2_ and the subsequent attachment of the termination groups T [34]. Monolayered structures are provided by transmission electron microscopy (TEM), element mapping is obtained by STEM-EDX mapping, and Fast Fourier Transform (FFT) of the Ti_3_C_2_ original image is observed (FEI Tecnai G2 F20, Hillsboro, OR, USA). The transmission electron microscopy (TEM) image of the monolayer Ti_3_C_2_ nanosheet is given in Figure 1d. The result reflects the single atomic layer feature of Ti_3_C_2_. High-resolution transmission electron microscopy (HRTEM) patterns and the dots painted on Figure 1e clearly show the crystalline lattice of the monolayer Ti_3_C_2_ nanosheet with a hexagonal structure. In this case, the projected interatomic distance is ~0.203 Å. The latter can be better seen in 0.4 nm, which clearly demonstrates the individual Ti atomic positions. The Fast Fourier Transform of the original image (FFT) pattern, which is presented in the bottom panel of Figure 1e, reveals the hexagonal symmetry structure. To further determine the composition of the obtained Ti_3_AlC_2_, energy-dispersive X-ray (EDX) analysis shows the presence of Ti, C, F, and O elements, which confirms the composition of the Ti_3_C_2_ nanosheet in Figure 1e. Notably, a high intensity of oxygen was detected in both EDX and in the XRD (Figure 1c) spectra of the Ti_3_C_2_ nanosheet. In addition to the inherent Ti-C bond, the Ti_3_C_2_ nanosheet is predominantly oxygen-terminated. The linear absorbance of Ti_3_C_2_ nanosheet aqueous solution was investigated at room temperature by a UV-vis spectrometer (TU-1901, Persee, Auburn, CA, USA). As shown in Figure 1f, two absorption peaks can be observed at 245 and 325 nm, respectively, and these results are expected. During the synthesis process, MXene is functionalized with different groups after etching out the A-element from its precursor ternary transition metal carbide (the so-called MAX phase). From Figure 1f, high absorption can be observed in the UV region ranging from 225 to 375 nm. This result can be attributed to the band-gap energy of the oxidized MXene, which can also be predicted by theoretical calculations [35,36].

### 3.2. The Nonlinear Absorption of the Ti_3_C_2_ Nanosheet

**Figure 2:**
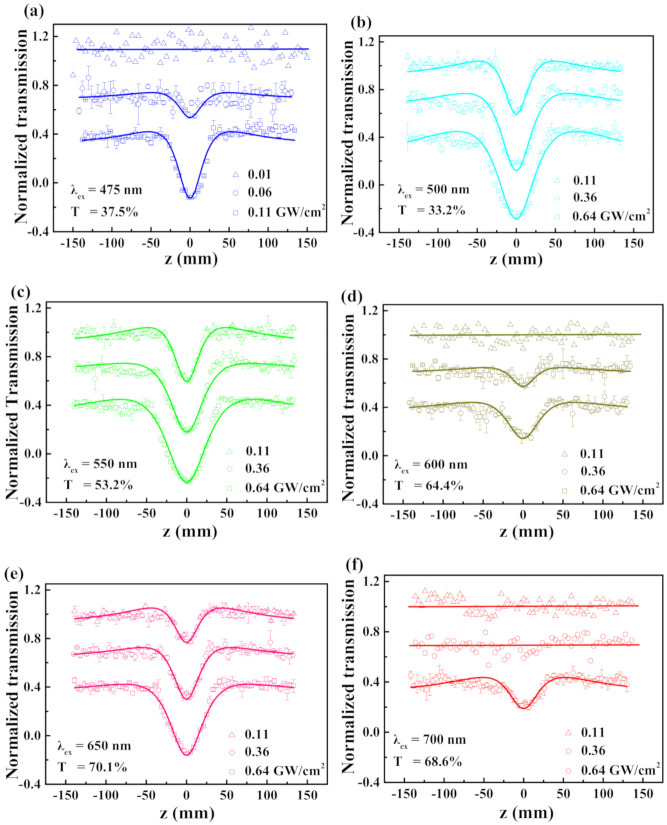
(**a**–**f**) Normalized transmission of the Ti_3_C_2_ nanosheet dispersion position for a nanosecond open aperture Z-scan under different wavelengths from 475 to 700 nm in the visible region, where the solid lines are the fitting curves. The fluence changes from 0.01 to 0.64 GW/cm^2^.

The OA Z-scan measurement results of Ti_3_C_2_ dispersions shown in Figure 2a–f was obtained at six laser wavelengths of 475, 500, 550, 600, 650, and 700 nm. In Figure 2a, when the incident pulsed laser energy is 0.01 GW/cm^2^, the normalized transmittance values of samples do not change, and no experimental signal is observed. In Figure 2a, as the pulsed laser energy increases to 0.06 GW/cm^2^, the transmittance values decrease with increasing energies. As the sample approaches the focal point (z = 0), a shallower valley is observed, indicating that reverse saturable absorption (RSA) occurs. In Figure 2a, when the energy increases to 0.11 GW/cm^2^, a deeper valley can be seen, indicating enhanced RSA. The experiment of Figure 2a proved that, at a lower energy, the phenomenon of RSA is not obvious, so we increased the incident energy to carry out the following experiments. With regards to Figure 2b,c, incident energies of 0.11, 0.36, and 0.64 GW/cm^2^ were used to carry out experiments. Interestingly, no detectable signals can be observed in Figure 2d, f at the low input energies. In Figure 2, the deepest valley of normalized transmittance is found at 550 nm, for the 0.64 GW/cm^2^ case, which implies that the nonlinear absorption of Ti_3_C_2_ is wavelength-dependent. In short, the Ti_3_C_2_ nanosheet exhibits broad-band RSA behavior, which means that it can be applied in optical limiting devices for the whole visible region. However, if we observe the results in Figure 2 carefully, we can find that the curves are not flat on either side of the central transmission dip, which means that a switch from saturable absorption (SA) to RSA occurs.

**Figure 3:**
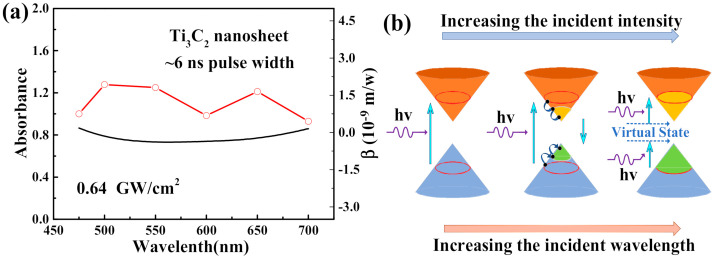
(**a**) The solid line shows the linear absorption spectra. The scatters are a theoretical fit of the nonlinear absorption coefficient of the Ti_3_C_2_ nanosheet as a function of wavelength. (**b**) A schematic diagram for reverse-saturable absorption processes.

As shown in Figure 3a, the nonlinear absorption coefficient of the Ti_3_C_2_ nanosheet as a function of the wavelength was obtained from Table 1. Linear absorption spectra are shown as a solid line. The linear absorption spectrum in Figure 3a showed that a one-photon absorption effect only occurred when the incident wavelength was less than 400 nm. The optical physical process can be visualized in Figure 3b [37]. When the irradiance was moderate, correlated with the light absorption, a large amount of electrons in Ti_3_C_2_ were pumped to an excited state, resulting in a small amount of electrons at the ground state, which is called the bleaching of ground state plasmon [22]. It is the bleaching of ground state plasmon that induces the SA. However, when the laser energy is increased further, electrons in the valence band can absorb two photons and jump to the conduction band, so two-photon absorption (TPA) occurs, which means that the sample shows RSA [38]. The nonlinear absorption in the Ti_3_C_2_ nanosheet was different from that of organic materials, in which the NLO performance originates from the effective combination of RSA and the photo-induced electron/energy transfer mechanism from donor to acceptor [39].

Based on the analysis above, combining the SA and the RSA, the total absorption coefficient can be written as follows [40]:

(1) $$\alpha (I)=\frac{\alpha_{0}}{1+(I/I_{s})}+\beta I,$$

where *α*_0_ is the linear absorption coefficient, *I* is the pulse laser intensity, and *I_s_* is the saturable intensity. For the purpose of eliminating the nonlinear effect from the solvents, the Z-scan measurement was conducted for the solvents at the same conditions without the Ti_3_C_2_ nanosheet. The normalized transmission of the open aperture Z-scan results can be described as follows [41]:

(2) T=∑m=0∞−q0(z)m(m+1)32≈1−βI0Leff22(1+z2z02).

In Equation (2), *β* is the nonlinear absorption coefficient; *I_0_* is the on-axis peak intensity at the focus; L is the sample length; *L_eff_* is the effective interaction length, and can be expressed as *L_eff_* = (1 − e^−α^_0_*^l^*)/*α*_0_; *z* is the longitudinal displacement of the sample from the focus (z = 0); and *z_0_* is the Rayleigh diffraction length.

In Figure 2, the theoretical fit conducted using Equations (1) and (2) is shown by solid lines, and the nonlinear absorption coefficient *β* was obtained and is listed in Table 1. We can observe that the TPA coefficients obtained at each wavelength increase with increasing laser irradiance. The largest values were recorded at 500 nm and the smallest ones were found at 700 nm. Such characters can be used in optical limiting devices to protect human eyes against optical damage.

### 3.3. Ultrafast Carrier Dynamics of the Ti_3_C_2_ Nanosheet

**Figure 4:**
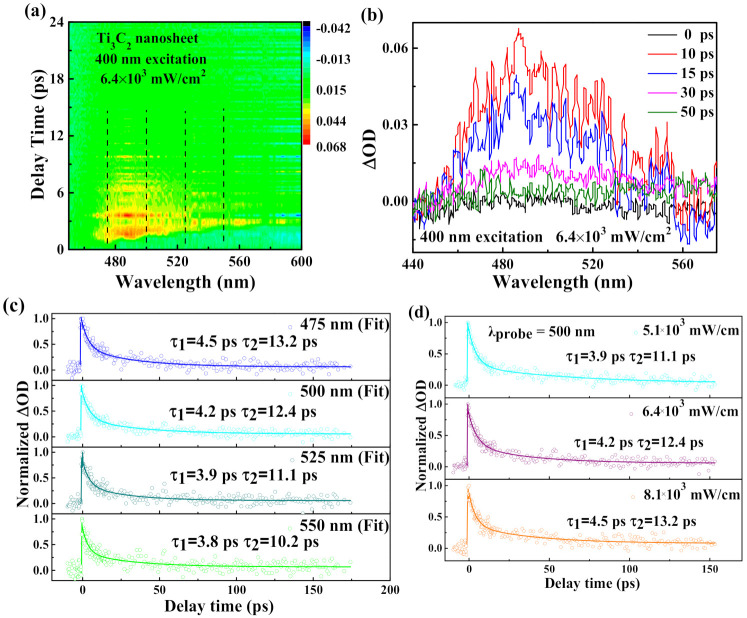
(**a**) Two-dimensional (2D) mapping of transient absorption spectra for the Ti_3_C_2_ nanosheet pumped at 400 nm with a fluence of 6.4 × 10^3^ mW/cm^2^. (**b**) Time and wavelength resolved transient absorption data of the Ti_3_C_2_ nanosheet under 400 nm excitation with 6.4 × 10^3^ mW/cm^2^. (**c**) Carrier dynamics (at 400 nm pumping) curves for the Ti_3_C_2_ nanosheet at different probe wavelengths of 475, 500, 525, and 550 nm respectively. The scatters are experimental data, while the solid lines are theoretically fit values generated with the pump fluence fixed at 6.4 × 10^3^ mW/cm^2^. (**d**) Carrier dynamics curves (at 400 nm pumping) at different pump fluences of 5.1 × 10^3^, 6.4 × 10^3^, and 8.1 × 10^3^ mW/cm^2^, with the probe wavelength fixed at 500 nm.

In order to study the ultrafast carrier dynamics of the Ti_3_C_2_ nanosheet, we performed broadband transient absorption measurement. In Figure 4a, the TA spectra of the Ti_3_C_2_ nanosheet are shown using an exemplary 2D map containing temporally and spectrally resolved TA signals. The 2D color-coded maps of broadband TA signals with a probe beam ranging from 450 to 600 nm were obtained at a constant pump fluence (6.4 × 10^3^ mW/cm^2^). The horizontal cut through the TA map lead to five differential absorption spectra at different delay times (0, 10, 15, 30, and 50 ps). In Figure 4b, a positive absorption indicates that excited state absorption (ESA) happened in the whole spectral region, and the ultrafast carrier relaxation process can be seen within the scale of ps. The amplitude of the TA spectrum obviously decreases with an increase of the delay time, which may be attribute to the carrier relaxation process of the Ti_3_C_2_ nanosheet. The curve of the 0 ps delay time (black) is the reference signal, when the Ti_3_C_2_ nanosheet was not excited. The peak of Ti_3_C_2_ appearing at the wavelength of 495 nm is attributed to photo-induced absorption which resulted from the transition process between occupied and unoccupied states. Additionally, this phenomenon was observed as RSA in the Z-scan experiment [42,43]. In ~50 ps, we can observe the TA signal relax to zero in the full waveband. In the TA measurement, the pump light is a 400 nm (~3.10 eV) laser whose energy is much higher than the energy bandgap of the Ti_3_C_2_ nanosheet (~2.04 eV). Therefore, after the excitation of the incident laser pulse, electrons are excited. The photoexcited electrons will jump to the conduction band with the time scale of tens of fs via the Franck–Condon transition, and the holes will stay in the valence band [44]. Then, the photoexcited carriers are quickly converted into hot carriers with a Fermi–Dirac distribution. Subsequently, the hot electrons will cool down as a fast relaxation process through electron–electron and electron–phonon scatterings on the conduction band. Ultimately, the hot electrons on the conduction band will relax back to the valence band and recombine with holes via carrier–phonon scatting through a different relaxation process.

In order to study the carrier dynamics at multiple wavelengths, normalized dynamic curves at the wavelength of 475, 500, 525, and 550 nm are given in Figure 4c. We can observe that the optical transmission responses of the Ti_3_C_2_ nanosheet consist of a fast decay component and a slow one. We ascribe the two components to their corresponding decay processes. After excitation, Coulomb-induced hot carriers, which are at the core state and trapped by the surface state, will release their spare energy through optical phonon scattering (3.9 ps). The other part of the cooled carriers will go through a nonradiative transition to the ground state within 30.1 ps [45,46]. The two different decays can be described by a biexponential decay function, which is expressed in Equation (3) [47].

(3) $$\frac{\Delta T}{T}=A_{1}\exp(-\frac{t}{\tau_{1}})+A_{2}\exp(-\frac{t}{\tau_{2}}),$$

where *A*_1_ and *A*_2_ refer to the amplitudes of each decay component. *τ_1_* and *τ_2_* are two parameters used to describe fast and slow decay lifetimes. As shown in Figure 4c by solid lines, the experimental data can be well-fitted by using Equation (3). As a result, the fast and slow relaxation components (*τ*_1_ and *τ*_2_) could be obtained and summarized. The two relaxation times *τ_1_* and *τ_2_* decrease with an increase of the probe wavelengths. This result can be attributed to electrons in the lower energy states being more likely be probed, which depopulated slower rather than higher energy states. An analogous phenomenon was observed in graphite [48].

Additionally, we also investigated the effect of pump fluence on the carrier dynamics at the probe wavelength of 500 nm. The experimental results obtained under three different pump fluences (5.1 × 10^3^, 6.4 × 10^3^, and 8.1 × 10^3^ mW/cm^2^) are shown in Figure 4d. By using Equation (3), the experimental data were well-fitted [47], and corresponding parameters were obtained.

With the increasing pump fluence, the observed lifetime of the fast decay component τ_1_ increases from 3.9 to 4.5 ps, and the slow decay component τ_2_ increases from 11.1 to 13.2 ps. The results are usually believed to originate from the carrier density dependence of electron–phonon coupling [49]. In general, the faster component of decay in 2D materials is attributed to the e–ph scattering and the slower one is related to phonon–phonon scattering [37]. The carrier–phonon interaction is a fundamental process during the energy transfer of the carrier [50]. As a result, the increased efficiency of the carrier–phonon interaction causes an increase of the cooling process. In other words, a high energy injection accelerates the relaxation process of carriers. A similar result has also been found in quantum dots and 2D films [51,52,53].

## 4. Conclusions

The NLO absorption properties of the Ti_3_C_2_ nanosheet were systematically studied via the OA Z-scan technique. The investigation reveals that, at a broadband visible range (475–700 nm), the Ti_3_C_2_ nanosheet exhibits an RSA property. Theoretical analysis indicates that the RSA mainly results from TPA. Furthermore, the ultrafast dynamics process of the sample was investigated using femtosecond transient absorption spectroscopy. The results imply that the relaxation of the process contains a fast decay component (~4 ps) and a slow one (~12 ps), and they are from electron–phonon and phonon–phonon interactions, respectively. Moreover, the two decay times increase with the pump fluence. The studies indicated that the Ti_3_C_2_ nanosheet can be used in ultrafast optoelectronics, such as optical limiters and novel photonic devices.

## Figures and Tables

**Table 1:** Nonlinear optical parameters of the Ti_3_C_2_ nanosheet.

λ (nm)	*I*_0_ (10^−2^ GW/cm^2^)	*β* (10^−9^ cm/mW)	λ (nm)	*I*_0_ (10^−2^GW/m^2^)	*β* (10^−9^ cm/mW)
475	0.10	-	500	0.74	1.03 ± 0.08
475	0.30	0.31 ± 0.04	500	1.10	1.32 ± 0.10
475	0.50	1.80 ± 0.11	500	1.40	1.93 ± 0.13
550	0.74	0.91 ± 0.06	600	0.74	-
550	1.10	1.12 ± 0.09	600	1.10	0.41 ± 0.05
550	1.40	1.81 ± 0.12	600	1.40	0.78 ± 0.06
650	0.74	0.84 ± 0.06	700	0.74	-
650	1.10	0.91 ± 0.07	700	1.10	-
650	1.40	1.65 ± 0.10	700	1.40	0.45 ± 0.04
